# Association of the New Peer Group–Stratified Method With the Reclassification of Penalty Status in the Hospital Readmission Reduction Program

**DOI:** 10.1001/jamanetworkopen.2019.2987

**Published:** 2019-04-26

**Authors:** Cian P. McCarthy, Muthiah Vaduganathan, Kershaw V. Patel, Hussain S. Lalani, Colby Ayers, Deepak L. Bhatt, James L. Januzzi, James A. de Lemos, Clyde Yancy, Gregg C. Fonarow, Ambarish Pandey

**Affiliations:** 1 Department of Medicine, Massachusetts General Hospital, Harvard Medical School, Boston; 2 Brigham and Women’s Hospital Heart & Vascular Center, Harvard Medical School, Boston, Massachusetts; 3 Division of Cardiology, Department of Internal Medicine, University of Texas Southwestern Medical Center, Dallas; 4 Division of Cardiology, Department of Medicine, Massachusetts General Hospital, Harvard Medical School, Boston; 5 Division of Cardiology, Northwestern University, Feinberg School of Medicine, Chicago, Illinois; 6 Division of Cardiology, Ronald Reagan UCLA Medical Center, Los Angeles, California

## Abstract

**Importance:**

Since the introduction of the Hospital Readmission Reduction Program (HRRP), readmission penalties have been applied disproportionately to institutions that serve low-income populations. To address this concern, the US Centers for Medicare & Medicaid introduced a new, stratified payment adjustment method in fiscal year (FY; October 1 to September 30) 2019.

**Objective:**

To determine whether the introduction of a new, stratified payment adjustment method was associated with an alteration in the distribution of penalties among hospitals included in the HRRP.

**Design, Setting, and Participants:**

In this retrospective cross-sectional study, US hospitals included in the HRRP for FY 2018 and FY 2019 were identified. Penalty status of participating hospitals for FY 2019 was determined based on nonstratified HRRP methods and the new, stratified payment adjustment method. Hospitals caring for the highest proportion of patients enrolled in both Medicare and Medicaid based on quintile were assigned to the low–socioeconomic status (SES) group.

**Exposures:**

Nonstratified and stratified Centers for Medicare & Medicaid payment adjustment methods.

**Main Outcomes and Measures:**

Net reclassification of penalties among all hospitals and hospitals in the low-SES group, in states participating in Medicaid expansion, and for 4 targeted medical conditions (acute myocardial infarction, heart failure, chronic obstructive pulmonary disease, and pneumonia).

**Results:**

Penalty status by both payment adjustment methods (nonstratified and stratified) was available for 3173 hospitals. For FY 2019, the new, stratified payment method was associated with penalties for 75.04% of hospitals (2381 of 3173), while the old, nonstratified method was associated with penalties for 79.07% (2509 hospitals), resulting in a net down-classification in penalty status for all hospitals by 4.03 percentage points (95% CI, 2.95-5.11; *P* < .001). For the 634 low-SES hospitals in the sample, the new method was associated with penalties for 77.60% of hospitals (492 of 634), while the old method was associated with penalties for 91.64% (581 hospitals), resulting in a net down-classification in penalty status of 14.04 percentage points (95% CI, 11.18-16.90; *P* < .001). Among hospitals that were not low SES (quintiles 1-4), the new payment method was associated with a small decrease in penalty status (1928 vs 1889; net down-classification, 1.54 percentage points; 95% CI, 0.38-2.69; *P* = .01). Among target medical conditions, the greatest reduction in penalties was observed among cardiovascular conditions (net down-classification, 6.18 percentage points; 95% CI, 4.96-7.39; *P* < .001).

**Conclusions and Relevance:**

The new, stratified payment adjustment method for the HRRP was associated with a reduction in penalties across hospitals included in the program; the greatest reductions were observed among hospitals in the low-SES group, lessening but not eliminating the previously unbalanced penalty burden carried by these hospitals. Additional public policy research efforts are needed to achieve equitable payment adjustment models for all hospitals.

## Key Points

**Question:**

Is the new, stratified payment adjustment method for the Hospital Readmission Reduction Program associated with an alteration in penalty distribution?

**Findings:**

This cross-sectional study of 3173 hospitals found that the new payment adjustment method was associated with a reduction in the proportion of hospitals penalized for fiscal year 2019, which corresponds to performance from July 1, 2014, to June 30, 2017, from 79.07% of hospitals (2509 hospitals) to 75.04% (2381 hospitals) compared with the old, nonstratified method. Hospitals with the largest share of patients of low socioeconomic status had the largest reduction.

**Meaning:**

The new payment adjustment method for the Hospital Readmission Reduction Program was associated with a more equitable distribution of penalties among hospitals, lessening the disproportionate burden carried by hospitals caring for patients of low socioeconomic status.

## Teaser

This cross-sectional study investigates whether the introduction of a new, stratified payment adjustment method is associated with an alteration in the distribution of penalties among hospitals included in the Hospital Readmissions Reduction Program.

## Introduction

Since the enactment of the Patient Protection and Affordable Care Act in 2010,^1^ several value-based programs have been established by the US Centers for Medicare & Medicaid (CMS), including the Hospital Readmission Reduction Program (HRRP).^2^ The objective of the HRRP is to lower CMS expenditures by reducing the burden of preventable repeated hospitalizations within 30 days while simultaneously improving the quality of postacute care. The program has been successful in reducing readmissions^3^; however, several concerns have emerged after its introduction.^4,5,6,7^ Risk adjustment models used to determine hospital penalty status under HRPP do not account for socioeconomic status (SES), which may influence hospital readmission risk but is not under the direct control of the health system.^7,8,9,10^ Since the establishment of the HRRP, penalties have fallen disproportionately on institutions that serve low-income populations, commonly referred to as *safety-net hospitals*.^11,12^ To address this concern, a component of the 21st Century Cures Act in 2016 mandated an adjustment in the HRRP to account for SES.^13^ In fiscal year (FY; October 1 to September 30) 2019, a new, peer group–based payment adjustment method was introduced that appropriates penalties within hospital systems that are deemed similar with respect to the SES of the patients they care for. Dual enrollment, the proportion of fee-for-service Medicare and Medicare Advantage hospitalizations for which the patient is eligible for both Medicare and full-benefit Medicaid, was chosen as the surrogate measure of socioeconomic disadvantage. To our knowledge, the impact of the new, peer group–based vs the old nonstratified HRRP method on the penalty status of participating hospitals has not been evaluated. Accordingly, we evaluated whether the new, stratified payment adjustment method was associated with an alteration in the distribution of penalties among hospitals included in the HRRP.

## Methods

### Nonstratified (Old) vs Stratified (New) CMS Payment-Adjustment Methods

Hospital performance under HRRP is assessed by calculating excess readmission ratios (ERRs) for 6 target conditions: acute myocardial infarction (AMI), heart failure (HF), pneumonia, chronic obstructive pulmonary disease (COPD), coronary artery bypass graft, and elective total hip and/or knee arthroplasty. The ERR is calculated as the ratio of predicted to expected readmissions for a given condition with 25 or more eligible discharges. Between FY 2013 and FY 2018, CMS used a nonstratified method of payment adjustment whereby a condition-specific ERR greater than 1.0 for a particular hospital was considered an indicator for performance below the national average for that condition. All hospitals were assessed using the same threshold of 1.0, and a payment adjustment factor formula accounting for all conditions with ERRs greater than 1.0 was used to calculate the size of the payment reduction. A maximum of a 3% reduction in CMS payments was levied at low-performing hospitals beginning in FY 2015.

A new peer group–based assessment of hospital performance was introduced in FY 2019. All eligible hospitals are now stratified into 5 peer groups (quintiles 1-5), based on the proportion of fee-for-service Medicare and Medicare Advantage hospital stays for which the patient is dually eligible for Medicare and full-benefit Medicaid. Hospital performance is assessed by comparing the condition-specific ERRs for each hospital with the median ERR of the peer group, thereby replacing the ERR threshold of 1.0 that was used for all hospitals under the nonstratified method. Similar to the nonstratified method, conditions with 25 or more eligible discharges and an ERR greater than the median ERR within the peer group are included in the payment adjustment factor formula to calculate the readmission penalty amount. The net payment reduction based on the readmission penalty for target conditions is capped at 3% under the new method, similar to the old method, and applies to all fee-for-service Medicare base operating diagnosis-related group payments for the FY.

### Setting and Participants

All hospitals that participated in the HRRP for FY 2019 were included in the analysis. We used publicly available CMS data to compare penalty status using the old and new payment adjustment methods under the HRRP, based on the ERR for 4 targeted medical conditions (AMI, HF, pneumonia, and COPD) for FY 2019 (performance period July 1, 2014, to June 30, 2017).^14^ Surgical conditions (coronary artery bypass graft and elective total hip and/or knee arthroplasty) were excluded from our analysis because of a lack of sufficient CMS-reported data. We compared the penalty status of both models in states stratified according to participation in Medicaid expansion. To do so, we identified states that adopted Medicaid expansion by the end of 2014.^15^ States with waivers were excluded from the Medicaid expansion group.

Hospital-level characteristics were obtained from American Hospital Association survey data from 2016 and linked to the publicly available data for the HRRP for FY 2019. This study was considered exempt from institutional review board or patient consent owing to use of publicly available hospital-level data. Individual patient-level data or characteristics were not accessed. This study followed the Strengthening the Reporting of Observational Studies in Epidemiology (STROBE) reporting guideline for cross-sectional studies.^16^

### Statistical Analysis

Net reclassification analysis was used to compare penalty status by the stratified and nonstratified payment adjustment methods for FY 2019. We examined the proportion of all hospitals that were up-classified (from nonpenalty to penalty status) and down-classified (from penalty to nonpenalty status) by the new stratified payment adjustment method. Characteristics of hospitals that were penalized by both methods, neither method, the old method only, or the new method only were compared using χ^2^ test. We then examined the net proportion of hospitals reclassified to a different penalty status by peer group quintile, by cardiovascular vs noncardiovascular condition, by targeted medical condition (AMI, HF, pneumonia, and COPD), and by state participation in Medicaid expansion during the study. Hospitals with the largest share of patients with dual-enrollment status for Medicare and Medicaid (quintile 5) were classified as the low-SES group.

Among the subgroup of hospitals that participated in the HRRP for FY 2018 (using the old payment adjustment method for performance period July 1, 2013, to June 30, 2016) and FY 2019, we performed sensitivity analyses to compare the penalty status across the 2 years based on the ERR for the 4 targeted medical conditions.^14,17,18^ The difference in the net payment reduction for participating hospitals based on the readmission penalty for all target conditions was also compared between FYs 2018 and 2019. All statistical analyses were performed using SAS version 9.1 (SAS Institute). All significance tests were 2-tailed, with *P* < .05 considered statistically significant.

## Results

### Reclassification of Penalty Status for All Hospitals in FY 2019

Penalty results for FY 2019 were available for 3173 hospitals. The new method divided hospitals into 5 quintiles based on the percentage of dual-eligible stays (eFigure 1 in the Supplement): quintile 1 (<13.70% dual-eligible stays), quintile 2 (13.70%-18.40%), quintile 3 (18.41%-23.23%), quintile 4 (23.24%-30.98%), and quintile 5 (>30.98%).

**Figure 1.**
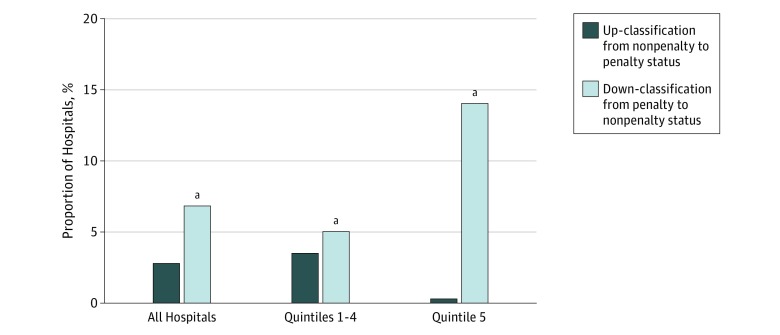
Reclassification of Penalty Status for All Hospitals According to Dual-Eligibility Quintile Based on Old and New Penalty Adjustment Methods for Fiscal Year 2019 Quintiles based on the percentage of dual-eligible stays: quintile 1, less than 13.70%; quintile 2, 13.70% to 18.40%; quintile 3, 18.41% to 23.23%; quintile 4, 23.24% to 30.98%; and quintile 5, greater than 30.98%. ^a^*P *<* *.001 compared with up*-*classification.

Using the old method, 79.07% of hospitals (2509 of 3173) would be subject to penalties for FY 2019. However, the new method resulted in a net down-classification in penalty status for all hospitals by 4.03 percentage points (95% CI, 2.95-5.11; *P* < .001), to 75.04% (2381 of 3173) (Table 1). The new peer group–based method up-classified 2.80% of hospitals (89 of 3173) (95% CI, 2.20%-3.40%; *P* < .001) to a penalty status but down-classified 6.83% (217 of 3173) (95% CI, 5.96%-7.70%; *P* < .001) to a nonpenalty status (Figure 1). Hospitals that were down-classified from penalized to nonpenalized were more commonly nonteaching and physician-owned hospitals, located in rural regions, with fewer hospital beds, less fully implemented electronic medical records, and limited cardiac surgery and percutaneous coronary intervention capabilities (Table 2). Hospitals up-classified to a penalty status were more likely to be teaching hospitals, participate in bundled-payment programs, and have cardiac surgery, percutaneous coronary intervention, and cardiac rehabilitation capabilities (Table 2).

**Table 1.** Net Down-Classification of Penalty Status for Hospitals Across Different Groups for Target Conditions Based on Old and New Penalty Adjustment Methods for Fiscal Year 2019

Condition	All Hospitals (N = 3173)		Hospitals That Are Not Low SES, Quintiles 1-4 (n = 2539)		Low-SES Hospitals, Quintile 5 (n = 634)		State Participation in Medicaid Expansion (n = 1317)		State Nonparticipation in Medicaid Expansion (n = 1856)	
	Net Down-Classification in Penalty Status, No. (%)	*P* Value	Net Down-Classification in Penalty Status, No. (%)	*P* Value	Net Down-Classification in Penalty Status, No. (%)	*P* Value	Net Down-Classification in Penalty Status, No. (%)	*P* Value	Net Down-Classification in Penalty Status, No. (%)	*P* Value
All conditions	128 (4.03)	<.001	39 (1.54)	.01	89 (14.04)	<.001	55 (4.18)	<.001	55 (3.07)	<.001
AMI	283 (8.92)	<.001	163 (6.42)	<.001	120 (18.93)	<.001	122 (9.26)	<.001	156 (8.72)	<.001
HF	67 (2.11)	<.001	−48 (−1.89)	.001	115 (18.14)	<.001	59 (4.48)	<.001	0	>.99
COPD	20 (0.63)	.16	−40 (−1.58)	<.001	60 (9.46)	<.001	14 (1.06)	.11	−1 (−0.06)	.93
Pneumonia	26 (0.82)	.10	−64 (−2.52)	<.001	90 (14.20)	<.001	22 (1.67)	.04	−7 (−0.39)	.54

Abbreviations: AMI, acute myocardial infarction; COPD, chronic obstructive pulmonary disease; HF, heart failure; SES, socioeconomic status.

**Table 2.** Hospital-Level Characteristics by Penalty Status Based on Old and New Penalty Adjustment Methods for Fiscal Year 2019

Characteristic^a^	No. (%)				*P* Value^b^
	Always Penalized	Down-Classified to No Penalty	Up-Classified to Penalty	Never Penalized	
No.	2292	217	89	575	NA
Hospital beds, median (IQR), No.^c^	176 (94-310)	65 (33-126)	129 (58-248)	100 (37-250)	<.001
Admissions, median (IQR), No.^c^	7698 (3561-14 779)	1977 (818-4676)	5381 (2429-11 020)	3849 (1210-11 823)	<.001
Total Medicare-days, median (IQR), No.^c^	18 729 (8135-35 497)	4462 (1501-11 508)	11 308 (4349-23 930)	7689 (1963-27 849)	<.001
Total Medicare discharges, median (IQR)^c^	3667 (1707-6635)	873 (374-2099)	2507 (1057-5171)	1796 (575-5656)	<.001
Adopted Medicaid expansion	1020 (44.9)	93 (46.7)	38 (42.7)	166 (30.2)	<.001
Organizational structure^d^					
For profit	522 (23.0)	55 (27.6)	14 (15.7)	155 (28.2)	<.001
Government	308 (13.6)	52 (26.1)	11 (12.4)	77 (14.0)	
Nongovernment, nonprofit	1440 (63.4)	92 (46.2)	64 (71.9)	317 (57.7)	
Hospital location^d^					
Rural	549 (24.2)	64 (32.2)	27 (30.3)	138 (25.1)	.06
Urban	1721 (75.8)	135 (67.8)	62 (69.7)	411 (74.9)	
Community hospital^d^	2263 (99.7)	186 (93.5)	89 (100)	539 (98.2)	<.001
Physician-owned hospital^c^	67 (3.5)	16 (12.3)	5 (6.4)	82 (17.8)	<.001
Electronic health records^d^					
Fully implemented	1442 (85.3)	90 (76.3)	65 (91.6)	362 (86.0)	.01
Partially implemented	239 (14.1)	25 (21.2)	5 (7.0)	54 (12.8)	
Cardiac surgery available^d^	831 (43.7)	15 (11.5)	30 (38.5)	186 (40.4)	<.001
Percutaneous coronary intervention available^d^	1186 (62.4)	22 (16.9)	44 (56.4)	233 (50.7)	<.001
Participation in bundle payment program^d^	464 (26.3)	14 (11.7)	20 (26.7)	112 (25.6)	.01
Cardiac rehabilitation available^d^	1353 (71.2)	42 (32.3)	52 (66.7)	291 (63.3)	<.001
Accredited by The Joint Commission^d^	1847 (81.4)	129 (64.8)	66 (74.2)	395 (72.0)	<.001
Teaching affiliation^d^					
Major	190 (8.4)	6 (3.0)	0	32 (5.8)	<.001
Minor	1005 (44.3)	66 (33.2)	37 (41.6)	217 (39.5)	
Nonteaching	1075 (47.4)	127 (63.8)	52 (58.4)	300 (54.6)	

Abbreviations: IQR, interquartile range; NA, not applicable.

^a^ Data on hospital-level characteristics were available for 3107 of 3173 hospitals included in the analysis.

^b^ *P* value calculated for all-group comparison.

^c^ Continuous variables compared with Kruskal-Wallis test across groups.

^d^ Categorical variables compared with χ^2^ test across groups.

### Reclassification of Penalty Status by SES in FY 2019

A total of 634 hospitals in quintile 5 were classified as being low-SES hospitals in FY 2019. Applying the old method, 91.64% of low-SES hospitals (581 of 634) would be subject to penalties in FY 2019. The new method resulted in a net down-classification in penalty status, with 77.60% (492 hospitals) subject to penalties, a 14.04–percentage point decrease (95% CI, 11.18-16.90; *P* < .001) (Table 1). Among the 2539 hospitals that were not low SES (quintiles 1-4 of proportion of dual-coverage patients), 75.94% (1928 of 2539) would be subject to penalties using the old method. The new method led to a small net down-classification in penalty status for these hospitals by 1.54 percentage points (95% CI, 0.38-2.69; *P* = .01) to 74.40% (1889 of 2539) (Table 1; Figure 1).

### Reclassification of Penalty Status for Targeted Medical Conditions in FY 2019

**Figure 2.**
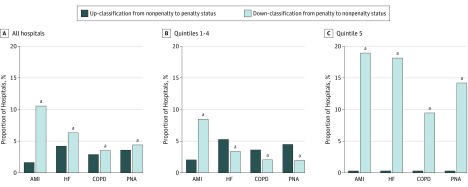
Reclassification of Penalty Status for Targeted Conditions Based on Old and New Penalty Adjustment Methodologies for Fiscal Year 2019 Quintiles based on the percentage of dual-eligible stays: quintile 1, less than 13.70%; quintile 2, 13.70% to 18.40%; quintile 3, 18.41% to 23.23%; quintile 4, 23.24% to 30.98%; and quintile 5, greater than 30.98%. AMI indicates acute myocardial infarction; COPD, chronic obstructive pulmonary disease; HF, heart failure; and PNA, pneumonia. ^a^*P* < .001 compared with up-classification.

Cardiovascular conditions (AMI or HF) would result in penalization of 63.41% of hospitals (2012 of 3173; 1359 for AMI; 1481 for HF) when applying the old method to FY 2019. The new model resulted in a net down-classification for 6.18 percentage points (95% CI, 4.96-7.39; *P* < .001), penalizing 57.23% of hospitals (1816 of 3173) for readmissions related to cardiovascular conditions (1076 of 3173 for AMI; 1414 for HF). In the overall cohort, the net down-classification in penalty status related to cardiovascular conditions was higher for AMI (8.9 percentage points; 95% CI, 7.73-10.11; *P* < .001) than for HF (2.1 percentage points; 95% CI, 0.98-3.24; *P* < .001) (Table 1; Figure 2).

Applying the old method to FY 2019, readmissions related to noncardiovascular conditions (COPD and pneumonia) would result in penalization of 63.57% of hospitals (1326 of 3173 for COPD; 1322 for pneumonia). In the overall cohort, there was no significant reclassification of hospitals’ penalty status by the new model for COPD and pneumonia for FY 2019 (COPD net down-classification: 0.63 percentage point; 95% CI, −0.27 to 1.53; *P* = .16; pneumonia net down-classification: 0.82 percentage point; 95% CI, −0.18 to 1.82; *P* = .10) (Table 1; Figure 2). Among the low-SES hospitals, there was a consistent down-classification in penalty status for all targeted medical conditions, with no up-classification in penalty status for any targeted condition (Table 1; Figure 2). In contrast, among hospitals not in the low-SES group, there was a significant net down-classification in penalty status for AMI. However, up-classification in penalty status occurred with HF, COPD, and pneumonia (Table 1; Figure 2).

### Reclassification of Penalty Status According to State Participation in Medicaid Expansion in FY 2019

Overall, 1317 hospitals (41.51%) in the cohort were in states that adopted Medicaid expansion by the end of 2014, of which 84.51% (1113 of 1317) would be subject to penalties using the old method for FY 2019. The new method resulted in a significant net down-classification in penalty status of 4.18 percentage points (2.89% [38 of 1317] up-classified to penalty status, 7.06% [93 of 1317] down-classified to nonpenalty status) among hospitals in states that adopted Medicaid expansion (Table 1; eFigure 2 in the Supplement). This was largely associated with the down-classification in penalty status driven by cardiovascular conditions, particularly AMI (Table 1). Similar patterns of reclassification were also noted among hospitals in states that did not participate in Medicaid expansion during the study period (Table 1; eFigure 2 in the Supplement).

### Reclassification of Penalty Status for Hospitals in FY 2019 (New Method) vs FY 2018 (Old Method)

The proportion of all hospitals subject to penalties by the HRRP for the 4 targeted medical conditions declined from 79.33% (2517 of 3173) in FY 2018 to 75.04% (2381 of 3173) in FY 2019 (net decrease, 4.3 percentage points; 95% CI, 2.95-511; *P* < .001). Hospitals of low SES had the largest decrease in penalties, from 92.74% (588 of 634) in FY 2018 to 77.60% (492 hospitals) in FY 2019 (net decrease, 15.1 percentage points; 95% CI, 11.90-18.39; *P* < .001). The proportion of hospitals not in the low-SES group that received penalties in 2018 and 2019 were numerically comparable; 74.40% (1889 of 2539) were subject to penalties in FY 2019 compared with 75.97% (1929 hospitals) in FY 2018 (eFigure 3 in the Supplement).

**Figure 3.**
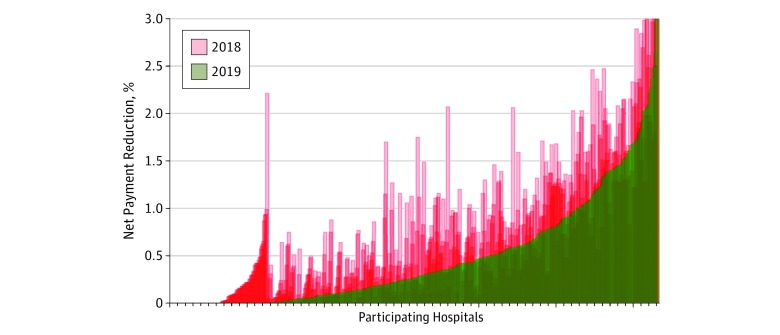
Net Payment Readjustment for All Target Conditions in Fiscal Year (FY) 2018 vs FY 2019 Among Hospitals in the Low–Socioeconomic Status Group Red boxes on the x-axis represent specific net payment reduction per hospital for FY 2018. The overlaying line graph in green shows net payment reduction in FY 2019. Median (interquartile range) payments for FY 2018 were 0.46% (0.14%-1.04%); median (interquartile range) payments for FY 2019 were 0.28% (0.04%-0.69%).

Examining the total amount of penalties imposed on hospitals in the low-SES group, the median (interquartile range) net payment reduction decreased from 0.46% (0.14% to 1.04%) in FY 2018 to 0.28% (0.04% to 0.69%) in FY 2019 (median change, −0.12%; interquartile range, −0.38% to 0%; *P* < .001) (Figure 3). Among hospitals that were not in the low-SES group, the median (interquartile range) net payment reduction increased modestly from 0.32% (0.03% to 0.85%) in FY 2018 to 0.35% (0.06% to 0.86%) in FY 2019 (median change, 0%; interquartile range, −0.12% to 0.17%; *P* = .01).

## Discussion

There were several noteworthy findings in our study, which examined the new, stratified peer group–based payment adjustment method for the HRRP. First, the down-classification to nonpenalty status was more commonly observed among hospitals that provide care for a disproportionately larger population of patients with socioeconomic disadvantage. Second, stratification was associated with a net down-classification in penalty status for hospitals included in the program. However, penalty rates remain high with the new method; 75% of all hospitals were subject to penalties for FY 2019. Third, among targeted medical conditions, the new HRRP penalty model was associated with a greater net down-classification for readmissions related to cardiovascular conditions than for readmissions related to COPD or pneumonia. Fourth, no major differences were seen in the reclassification of penalty status in hospitals stratified by state participation in Medicaid expansion. Our findings were consistent when comparing old and new methodologies for FY 2019 and when comparing the actual penalties applied to hospitals by the old method in FY 2018 with the new method in FY 2019.

Hospitals that were down-classified to nonpenalty status were more often small, nonteaching, and physician owned, located in rural areas, and had the highest proportion of patients with dual-enrollment status, a strong predictor of a higher burden of socioeconomic risk factors.^19^ Lower-income patients tend to have worse health outcomes on many performance quality measures, and hospitals that disproportionately serve this patient population are more likely to identify as poor performing and are subject to higher penalties in traditional value-based payment programs.^20^ However, multiple factors that are not related to the quality of care received, such as greater disease severity, lack of social support, and poor living environment, may underlie the poor clinical outcomes among patients with socioeconomic risk factors. Performance-based penalties incurred by the hospitals that predominantly care for low-income patients may take away important financial resources that are needed to care for these patients with the most complex social and medical needs. Furthermore, it may also disincentivize hospitals from taking care of such patients. Findings from our study suggest that the new payment adjustment method for the HRRP may contribute to a more socially equitable implementation of readmission performance–based penalties. We observed the largest down-classification in penalty status among hospitals in the low-SES group that were previously disproportionately penalized by the old, nonstratified adjustment method.^11^ For these hospitals, the new system was associated with a down-classification in penalty risk status across all conditions without any up-classification. We also observed consistent patterns of reclassification in penalty status under the new method in states with and without expansion of the Medicaid program. This suggests that the down-classification in hospital penalty status may not be related to individuals who newly obtained Medicaid.

Cardiovascular conditions had consistently greater down-classification in penalty status compared with noncardiovascular conditions. The highest net down-classification across individual target conditions was observed with AMI, which has a particularly high financial burden among low-income patients.^21^ Furthermore, hospitals that were down-classified to nonpenalty status were less likely to have cardiac surgery or percutaneous coronary intervention available, reflecting the socioeconomic disparities that exist in access to cardiovascular care.

The new stratified method for assigning penalty status has several advantages. First, it recognizes the greater challenges that hospitals that care for a higher proportion of patients of low SES face in achieving high performance. Second, it may attenuate concerns that penalizing safety-net hospitals for performance could lead to worse access to care for patients of low SES. Third, failing to adjust for socioeconomic differences in populations may lead to inaccurate representation of the quality of care provided to patients by hospitals. Nevertheless, the recalibration of penalties to a peer group–based method reduces but does not eliminate the disproportionate penalty burden.

The new method accounts for some of the socioeconomic disparities across hospitals; however, the challenges related to incomplete adjustment for disease severity remain unaddressed. For instance, while small, rural, low-SES hospitals benefit from the new stratified method, large, urban safety-net hospitals remain at risk of disproportionate penalties. Taken together, the stratified peer group–based assessment of readmission-based hospital performance is a welcome initial step, but more work is needed, with further adjustments that might more capably capture social determinants of health beyond SES.

It is important to recognize that hospital performance at low-SES hospitals is influenced by factors other than just patient characteristics. Examining patients who had 2 admissions for similar diagnoses to hospitals in the best- and worst-performing quartiles for hospital readmission performance, Krumholz et al^22^ found that readmission rates were higher when the same patient was admitted to the worst-performing hospitals compared with the best-performing hospitals, suggesting that hospital quality is, in part, contributing to readmission rates independent of patient characteristics. Additionally, in the first 3 years of the HRRP, safety-net hospitals reduced their readmissions for AMI, HF, and pneumonia.^23^ Whether such reductions were driven by improvement in hospital quality, changes in triage patterns, or upcoding of diagnoses is unknown.^24^ It is also possible that increased resources were needed to improve quality and reduce readmissions at safety-net hospitals, which could be shifting resources from other necessary areas of clinical care. Future studies are also needed to determine if the new payment adjustment method will lead to meaningful changes in hospital behavior, fewer unintended consequences, and potentially improved health outcomes, such as mortality and readmission rates. Prospective accounting of global system costs incurred by patients, hospitals, and payers is needed to determine the potential economic impact of the new payment adjustment method.

### Limitations

Our study has several limitations. First, there is no single, agreed-on method to identify hospitals that care for patients of low SES. In our study, the hospitals with the highest proportion of dual-eligible patients were considered high-socioeconomic-risk hospitals. This is consistent with findings from prior studies suggesting that the proportion of dual-eligibility patients is a robust metric in accounting for socioeconomic risk^25^ and most closely reflects the new method of peer group comparisons. Second, given insufficient data, we did not account for surgical target conditions in the penalty status adjustment estimates based on the old and new systems. Third, some of the changes in penalty status from FY 2018 to FY 2019 may also have been related to implementation of performance improvement measures and not just changes in the HRRP method.

## Conclusions

The new, stratified penalty adjustment method for the HRRP was associated with a reduction in penalties across hospitals included in the program; the greatest reductions were observed among hospitals in the low-SES group. This reduces the previously unbalanced penalty burden carried by these hospitals to some extent. Although this payment adjustment scheme appears to improve the equitable distribution of hospital penalties, 75% of hospitals were subject to penalties in FY 2019. Thus, the new peer group–based penalty model represents a step in the right direction. Sustained efforts are needed to better account for social determinants of health in hospital performance models and to allow for more socially just and equitable distribution of performance-based financial incentives.

## Notes

Supplement. **eFigure 1. **Distribution of Proportion of Dual Eligibility for Medicare and Full-Benefit Medicaid Across Hospitals **eFigure 2. **Reclassification of Penalty Status Stratified by Medicaid State Expansion for Fiscal Year 2019 **eFigure 3. **Reclassification of Penalty Status From Fiscal Year 2018 (Old Methodology) to Fiscal Year 2019 (New Methodology)
