# Supplementary material for: Association of the New Peer Group–Stratified Method With the Reclassification of Penalty Status in the Hospital Readmission Reduction Program
Source: JAMA Netw Open. 2019 Apr 26;2(4):e192987. doi: 10.1001/jamanetworkopen.2019.2987 (PMC6487568; doi:10.1001/jamanetworkopen.2019.2987)
Supplement: Supplement. — eFigure 1. Distribution of Proportion of Dual Eligibility for Medicare and Full-Benefit Medicaid Across Hospitals eFigure 2. Reclassification of Penalty Status Stratified by Medicaid State Expansion for Fiscal Year 2019 eFigure 3. Reclassification of Penalty Status From Fiscal Year 2018 (Old Methodology) to Fiscal Year 2019 (New Methodology) [file jamanetwopen-2-e192987-s001.pdf]

Supplementary Online Content

McCarthy CP, Vaduganathan M, Patel KV, et al. Association of the new peer group–stratified method with the reclassification of penalty status in the Hospital Readmission Reduction Program. *JAMA Netw Open*. 2019;2(4):e192987. doi:10.1001/jamanetworkopen.2019.2987

eFigure 1. Distribution of Proportion of Dual Eligibility for Medicare and Full-Benefit Medicaid Across Hospitals

eFigure 2. Reclassification of Penalty Status Stratified by Medicaid State Expansion for Fiscal Year 2019

eFigure 3. Reclassification of Penalty Status From Fiscal Year 2018 (Old Methodology) to Fiscal Year 2019 (New Methodology)

This supplementary material has been provided by the authors to give readers additional information about their work.

© 2019 McCarthy CP et al. *JAMA Network Open.*

eFigure 1. Distribution of Proportion of Dual Eligibility for Medicare and Full-Benefit Medicaid Across Hospitals

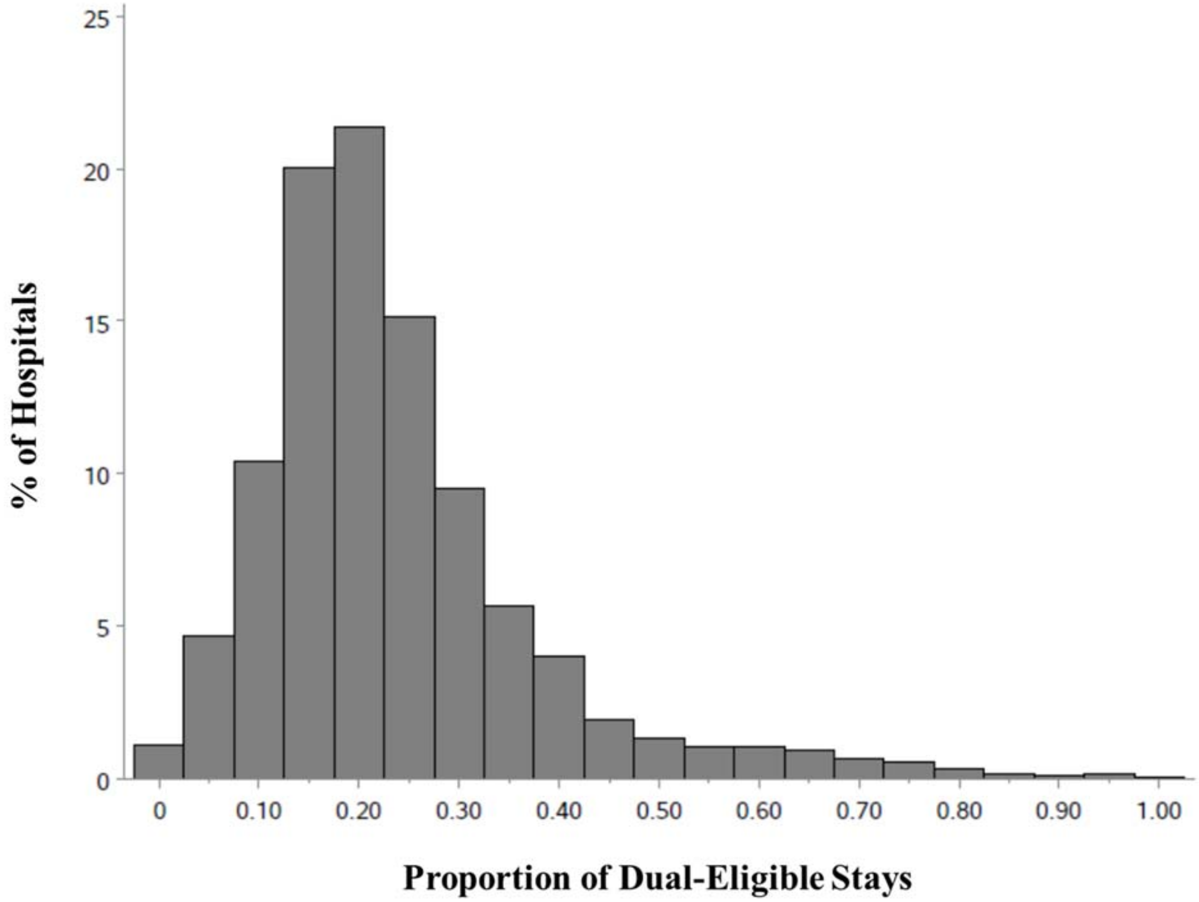

© 2019 McCarthy CP et al. *JAMA Network Open.*

eFigure 2. Reclassification of Penalty Status Stratified by Medicaid State Expansion for Fiscal Year 2019

Proportion of hospitals with up- (from non-penalty to penalty status) & down- (from penalty to non-penalty) classification of penalty status with the new peer-group based performance metric across groups stratified by states with vs. without expansion of the Medicaid Program (before end of 2014).

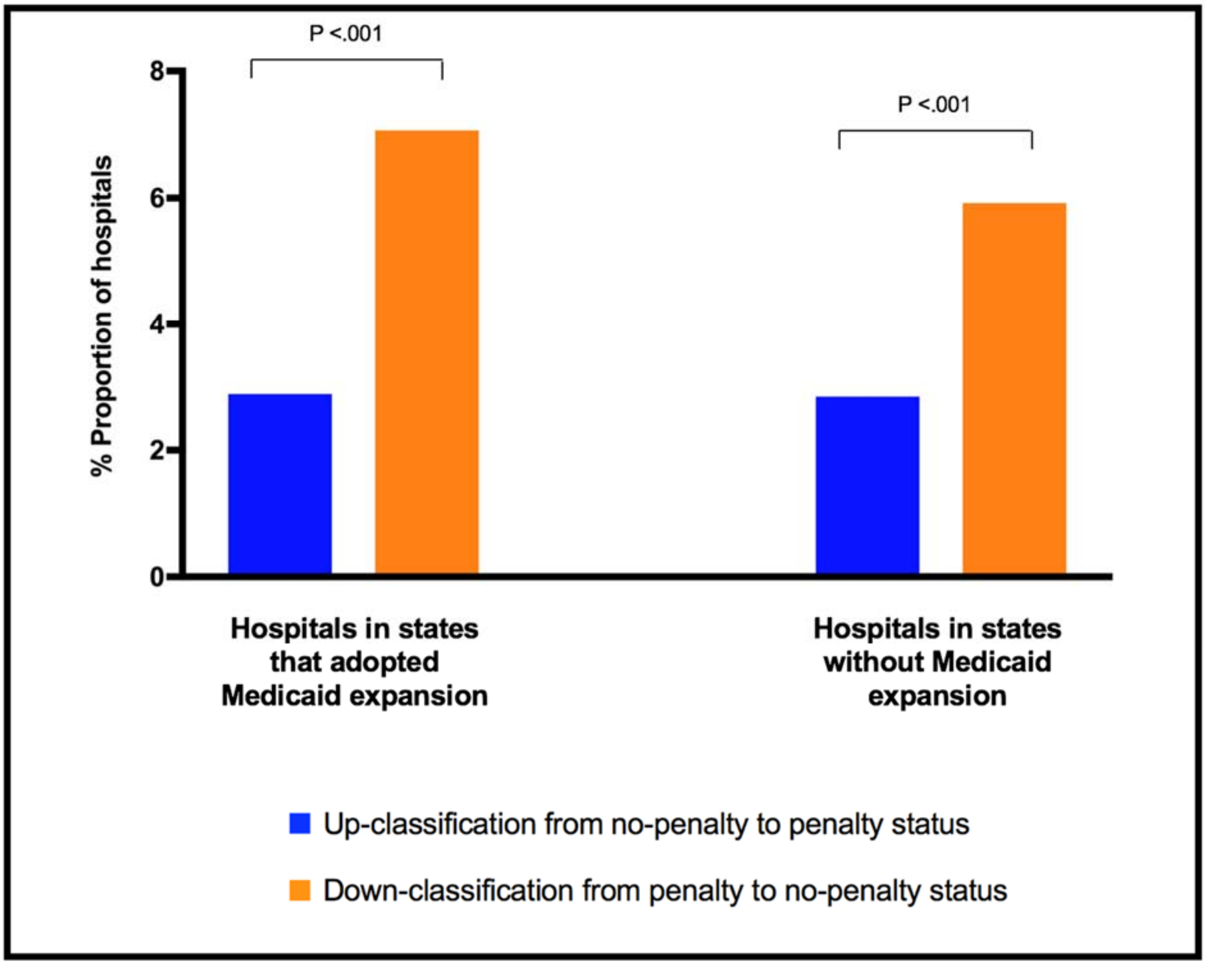

© 2019 McCarthy CP et al. *JAMA Network Open.*

eFigure 3. Reclassification of Penalty Status from Fiscal Year 2018 (Old Methodology) to Fiscal Year 2019 (New Methodology)

Proportion of hospitals with up- (from non-penalty to penalty status) & down- (from penalty to non-penalty) classification of penalty status with the new peer-group based performance metric (Fiscal Year 2018 versus Fiscal Year 2019).

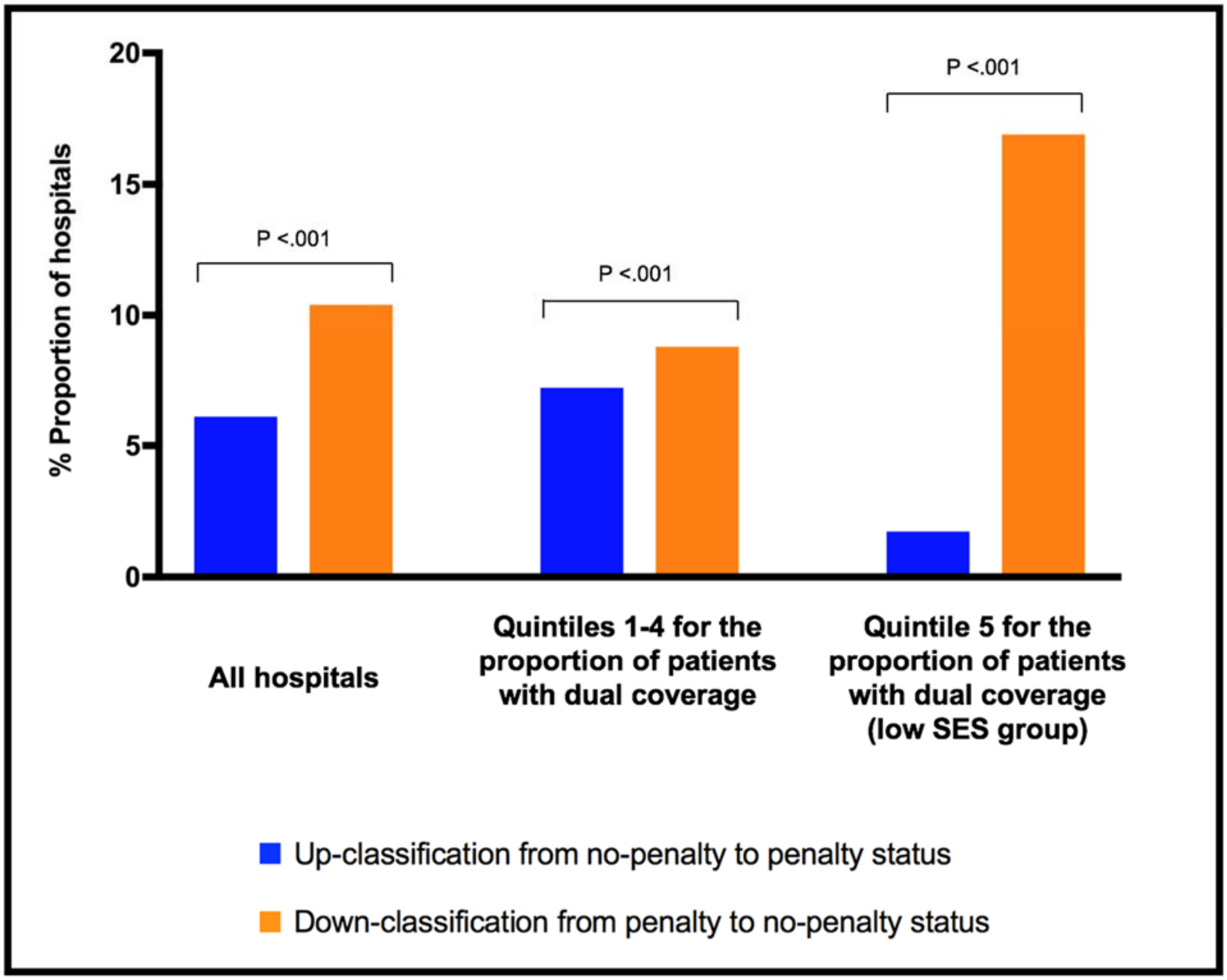

© 2019 McCarthy CP et al. *JAMA Network Open.*
